# Can mitochondria brown the lower-limb adipocytes?

**DOI:** 10.1016/j.omtm.2023.101181

**Published:** 2024-01-05

**Authors:** Ilias P. Doulamis, Aspasia Tzani

**Affiliations:** 1 Department of Surgery, Lahey Hospital and Medical Center, Burlington, MA, USA; 2 Department of Cardiac Surgery, Boston Children’s Hospital, Harvard Medical School, Boston, MA, USA; 3 Brigham and Women’s Hospital Heart and Vascular Center, Harvard Medical School, Boston, MA, USA

Lower limb ischemia reperfusion injury (IRI) is commonly observed in the clinical setting either in an unexpected urgent manner such as embolism, acute thrombosis, and trauma or electively, where it is iatrogenically caused for lower extremity revascularization surgery. Mitochondria exert a primary role in muscle function and, subsequently, in its recovery following such an insult.^1^ There is a plethora of studies suggesting that mitochondrial transplantation affords protective properties against IRI in the heart, kidneys, lungs, and skeletal muscle.^2^ A previous study has shown its efficacy in reducing skeletal muscle injury and improving hindlimb function and viability.^1^ However, understanding the mechanism of action through which mitochondrial transplantation exerts its protective role in lower-limb IRI is important. In this article, Zeng and co-workers investigated the therapeutic influence of human umbilical cord mesenchymal stem cell (hMSC)-derived mitochondria in a lower IRI model. They found that mitochondria were not only captured by skeletal muscle cells but also internalized in adipocytes, leading to its browning via optic atrophy 1 (OPA1)/uncoupling protein 1 (UCP1).^3^

A well-established model of lower-limb IRI was used, and mitochondria were isolated from hMSCs, which have been described as a source of mitochondria in previous studies.^4^ Further sources of mitochondria such as autologous skeletal muscle could be also opted for given it is also well studied and can simplify the procedure in a clinical setting. Healthy non-ischemic muscle would be the ideal source; however, studies from donation after cardiac death models have shown that briefly ischemic muscle tissue is not inferior to non-ischemic tissue.^5^

The authors assessed the integrity, viability, and count of mitochondria with immunofluorescence and electron microscopy, which are gold-standard techniques in preclinical studies.^5^ The identification of real-time assessment of mitochondria function and number without compromising their potential use is still an area of work in progress. Direct assessment of their number under a light microscope has been proposed in the clinical setting; however, it lacks the ability to further characterize mitochondrial quality.^2^

The most direct route of administration, which is direct injection to the muscle, was elected in this study. An alternative route would be administration through the femoral artery, which can be useful, especially when access to the whole muscle of interest is not available. This could be achieved either percutaneously or via a surgical cut down if that is needed, regardless of the setting of the surgery.^2^

An adequate follow-up period and functional assessment of the hindlimb were also established by the authors, providing further evidence to support their findings. Future studies uncovering the adipocyte-dependent effect on angiogenesis, especially on capillary formation and arterialization, will add significant value to those findings and will provide a deeper understanding on the underlying mechanisms. Paracrine effects on pericytes or adipocyte-derived exosomes directly affecting endothelial cells could potentially explain the beneficial effect of mitochondrial transplantation on the blood flow recovery of the hindlimb.

The novelty of this study is that it was observed that the transplanted mitochondria are being uptaken not only by skeletal muscle cells but by adipocytes too. This was shown by localization of the mitochondria in the adipocytes by immunofluorescence. UCP1 and OPA1 were also upregulated, suggesting that browning of the adipose tissue is associated with mitochondrial transplantation. To further support their hypothesis, they performed *in vitro* experiments, which showed that mitochondrial transplantation promoted UCP1 expression and adipocyte browning, but this effect was attenuated when OPA1 expression was silenced. Similarly, when UCP1 expression was silenced, the browning of the adipose tissue initially observed after mitochondrial transplantation was no longer evident. These *in vitro* findings will have to be further validated *in vivo* with knockout UCP1 and OPA1 animal models in order to assess the specific role of OPA1 and UCP1 in the observed adipocyte browning effect as suggested by the authors.

Overall, this article provides insight into a new aspect of mitochondrial transplantation in providing protection against lower-limb IRI: adipose tissue browning. This piece of information is of paramount importance for further applications of this therapeutic modality since recognizing its off-target effects can expand its therapeutic potential.
